# Ten Years of High Resolution Structural Research on the Voltage Dependent Anion Channel (VDAC)—Recent Developments and Future Directions

**DOI:** 10.3389/fphys.2018.00108

**Published:** 2018-03-07

**Authors:** Kornelius Zeth, Ulrich Zachariae

**Affiliations:** ^1^Department for Science and Environment, Roskilde University, Roskilde, Denmark; ^2^School of Science and Engineering, University of Dundee, Dundee, United Kingdom; ^3^School of Life Sciences, University of Dundee, Dundee, United Kingdom

**Keywords:** VDAC, structural biology, x-ray, NMR, Tom40

## Abstract

Mitochondria are evolutionarily related to Gram-negative bacteria and both comprise two membrane systems with strongly differing protein composition. The major protein in the outer membrane of mitochondria is the voltage-dependent anion channel (VDAC), which mediates signal transmission across the outer membrane but also the exchange of metabolites, most importantly ADP and ATP. More than 30 years after its discovery three identical high-resolution structures were determined in 2008. These structures show a 19-stranded anti-parallel beta-barrel with an N-terminal helix located inside. An odd number of beta-strands is also shared by Tom40, another member of the VDAC superfamily. This indicates that this superfamily is evolutionarily relatively young and that it has emerged in the context of mitochondrial evolution. New structural information obtained during the last decade on Tom40 can be used to cross-validate the structure of VDAC and vice versa. Interpretation of biochemical and biophysical studies on both protein channels now rests on a solid basis of structural data. Over the past 10 years, complementary structural and functional information on proteins of the VDAC superfamily has been collected from *in-organello, in-vitro*, and *in silico* studies. Most of these findings have confirmed the validity of the original structures. This short article briefly reviews the most important advances on the structure and function of VDAC superfamily members collected during the last decade and summarizes how they enhanced our understanding of the channel.

## Introduction

Gram-negative bacteria, mitochondria, and chloroplasts are enveloped by two lipid bilayers, termed the inner and outer membrane. While all inner membrane proteins are alpha-helical, proteins in the outer membrane display beta-barrel structures with a wide variation in the number and tilt of beta-strands as well as the way the strands are interconnected by loops and turns (Fairman et al., 2011). The mitochondrial porin VDAC (voltage-dependent anion channel) is the major protein in the mitochondrial outer membrane (MOM). It confers a sieve-like structure to the outer membrane due to its high abundance, covering about ~30% of the membrane surface (Gonçalves et al., 2007). The high density of VDAC in the outer membrane is surprising, but may be explained by the wide range of functions performed by VDAC isoforms in metabolite exchange and their interactions with proteins of the cytoplasm and the intermembrane space (Lemasters and Holmuhamedov, 2006). In particular, hexokinase-VDAC interactions were shown to dominate on the mitochondrial surface with a high surface density of this complex, including clusters of hVDAC3 isoforms with hexokinase I (Neumann et al., 2010). Further important interactions of VDAC at the mitochondrial surface are those undergone with pro- and anti-apoptotic proteins such as Bax, Bak, or tBid (Rostovtseva and Bezrukov, 2008; Ott et al., 2009).

**Figure 1:**
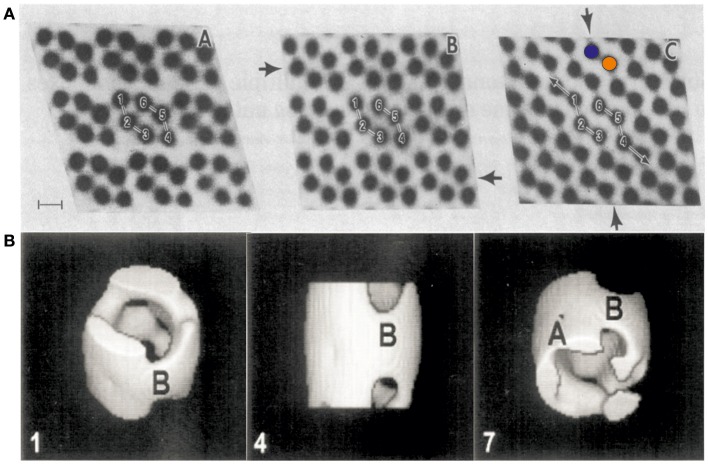
First electron micrographs of VDAC from the early 1980s. **(A)** Electron-microscopic investigations of ncVDAC arranged in isolated native membrane vesicles show three two-dimensional molecular arrays: two slightly different hexameric arrangements of VDAC pores around a two-fold symmetry axis (right and left) and another arrangement where dimeric VDAC pores form chain-like superstructures (colored circles mark two independent monomers) (Mannella et al., 1983). **(B)** A single particle analysis of small membrane arrays yielded the first 3D representation of VDAC at a resolution of ~2 nm (Guo et al., 1995). Figures reproduced with permission.

VDAC can form semi-crystalline arrays in the MOM at high protein concentration (Mannella et al., 1983; Gonçalves et al., 2007) (see Figures 1, **3B**). Early studies by electron microscopy (EM), performed in the laboratories of Frank and Mannella in the 1980s, yielded structural information on semi-crystalline arrays of a pore-forming channel isolated from the MOM (Mannella et al., 1983) (see Figures 1A,B). These studies showed hexagons of two channel triplets related by two-fold symmetry with a channel diameter of ~4 nm. Later studies of VDAC using electron microscopy in combination with single particle analysis revealed the 3D shape and dimensions of the channel at medium resolution (Guo et al., 1995). It took until 2008 however to unravel the structure of VDAC at atomic resolution.

## Three high-resolution structures of VDAC independently confirm an unexpected fold

**Figure 2:**
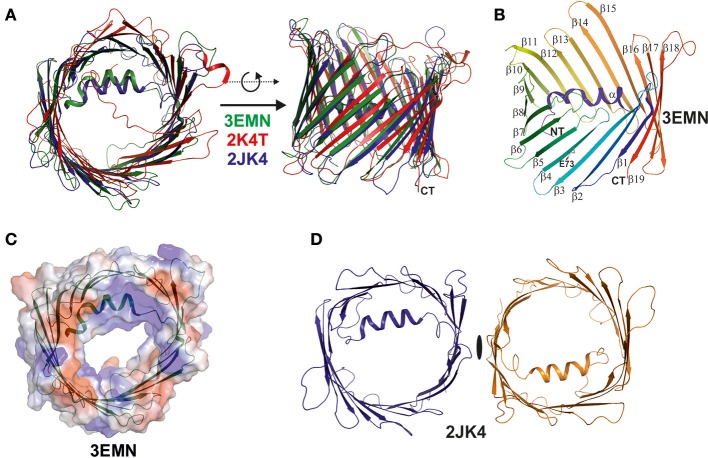
Superposition of the three VDAC structures published simultaneously in 2008. The three structures were determined by X-ray, NMR spectroscopy, and a combination of both methods (Bayrhuber et al., 2008; Hiller et al., 2008; Ujwal et al., 2008). **(A)** Here, we superimposed the three structures and displayed them in ribbon representation to highlight their analogies and differences. The X-ray structure (3EMN) is shown in green, the NMR structure (2K4T) is shown in red, while the hybrid structure (2JK4) is displayed in blue. The structures are viewed from two different perspectives related by a 90° rotation around the x-axis. The major difference between the structures is the location and secondary structure assigned to the N-terminal helix in the NMR structure. **(B)** Structure of mVDAC displaying the fold of the VDAC superfamily proteins. The structure is color coded from the N- (blue) to the C-terminus (red) and secondary structure elements are annotated (alpha, beta1-beta19). **(C)** A primary function of VDAC in the MOM is to translocate nucleotides and the surface representation of VDAC color coded in surface charge potentials is provided. This representation shows the channel pore together with the electrostatic surface potential which is primarily positive around the channel eyelet. **(D)** The crystal structure of 2JK4 showed a potential VDAC dimer in the crystal lattice and although the dimer contacts are rather weak, the interface formed by strands beta1 and beta19 appears to be biologically important. All figures were prepared using PYMOL (www.pymol.org).

In 2008, the simultaneous structure determination of VDAC in three independent laboratories, based on NMR spectroscopy and X-ray crystallography, provided a dramatically enhanced view on the architecture of VDAC (see Figures 2A,B) (Bayrhuber et al., 2008; Hiller et al., 2008; Ujwal et al., 2008). These studies were the first to reveal the structure of a member of the small class of proteins located within the MOM. They raised particular interest in the community for two further reasons, one of which was the discovery of the precise VDAC fold, while the second was to unravel the structural deviation from porins of Gram-negative bacteria (Bayrhuber et al., 2008; Zeth and Thein, 2010; Bay et al., 2012). The structures confirmed the dimensions of the VDAC pore previously observed by electron microscopy and revealed important further structural features, such as the 19-stranded nature of the channel, the presence of an alpha-helix located inside the pore and the strong internal positive charge (see Figures 2B,C) (Bayrhuber et al., 2008; Hiller et al., 2008; Ujwal et al., 2008). Additional structural information included the dimeric assembly of the protein reported by Bayrhuber et al. (2008), which resembled the oligomeric species revealed by the early EM data (see Figures 1C, 2D). Furthermore, the tilt of the beta-sheets, the length and orientation of surface-exposed loops and turns, and the electrostatic properties of VDAC were unraveled. It was also shown that E73, a residue potentially critical for apoptosis, unexpectedly faces toward the membrane environment (Villinger et al., 2010; Shoshan-Barmatz et al., 2017). Although the three structures differed in some details (for instance, the NMR structure did not fully resolve the alpha-helix and rather assigned a random coil structure in this area), the number and tilt of beta-strands and overall dimensions were clearly identical. Even though these structures were initially placed into doubt, due to the generation of the proteins by recombinant techniques followed by refolding, they were considered to be a breakthrough for the understanding of the MOM and their correctness was never challenged in the field of structural biology (Colombini, 2009; Hiller and Wagner, 2009).

## Structural and functional studies on VDAC and Tom40 confirm the VDAC-like fold *in vivo* and provide new mechanistic details

**Figure 3:**
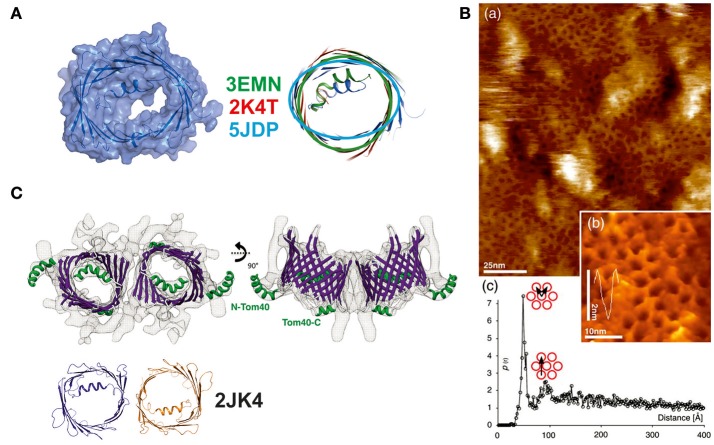
New structural details collected on members of the VDAC-superfamily of 19-stranded β-barrels. **(A)** Structure determination of the E73V mutant of human VDAC. This mutant was previously described to have altered biophysical properties in comparison to the wildtype protein. The NMR determination of the structure yielded a structurally significantly changed beta-barrel with an oval rather than a circular form and a smaller channel diameter. Three structures of the mVDAC1 (in green), hVDAC1 (in red), and the mutant structure (in light blue) were superimposed to show the deviation in the barrel section. A surface representation of the latest NMR structure (5JDP) shows the particular small diameter of the pore eyelet in E73V (Jaremko et al., 2016). **(B)** (a) High resolution AFM topographs of densely packed scVDAC natively embedded in membranes of *S. cerevisiae* (large picture) and (b) high resolution view of VDAC molecules in an arrangement similar to the early electron microsocopy images (inset picture). (c) Peak analysis of the separation between two VDAC molecules yields a distance of 53 Å (pore to pore; see graph) (Gonçalves et al., 2007). **(C)** Structural analysis of the Tom40 complex shows a dimeric arrangement of the pore structures with the alpha-helix inside the barrel or exposed to the surface. The structure at 1 nm resolution was modeled by a Tom40-homology model based on the mVDAC structure (PDB-entry 3EMN) and can accommodate the 19 beta-strands unambiguously. The dimeric arrangement is mediated by the same beta1/beta19 strands as previously reported for the hVDAC1 dimer (2JK4—dimer shown in orange and dark blue) (Bayrhuber et al., 2008; Bausewein et al., 2017). Figures reproduced with permission.

During the last decade, new studies offering refined structural and functional insights on VDAC have been conducted in the fields of biochemistry, structural, and computational biology (see Table 1). In a recent study of hVDAC1, high-resolution NMR spectroscopy was applied to determine the structure of the E73V mutant (Jaremko et al., 2016). Notably, and as previously mentioned, residue E73 has the unusual location at the outer face of the beta-barrel, with its side-chain pointing toward the membrane (Bayrhuber et al., 2008; Hiller et al., 2008; Ujwal et al., 2008). The N15 NMR data acquired returned a model that shows a strongly distorted beta-barrel relative to the mVDAC1 and hVDAC1 structures (a substantial r.m.s.d of ~3 Å for the Cα atoms after structure superposition) with an unusually narrow pore diameter (see Figure 3A). While this study primarily aimed at the development of NMR techniques in the context of a large membrane protein, a potentially altered function of the artificially constricted barrels remains conceivable. Another structural study published recently presents structures of hVDAC1 which were solved using protein produced in an *E. coli* cell-free expression system, but lacking the denaturation step previously applied to all VDAC preparations (Hosaka et al., 2017). This protein yielded the archetypical monomeric structure but showed two different crystal packings based on weak protein-protein interactions. The authors speculate that this feature might represent a potential binding interaction which may be important to form mixed oligomers of VDAC isoforms in membranes (Hosaka et al., 2017).

**Table 1:** 3D structures of VDAC published since 2008.

**Isoform**	**Species**	**Modification**	**Method**	**Oligomerization**	**Resolution (Å)**	**PDB code**	**References**
zfVDAC2	Zebra fish	Wildtype	X-ray	Dimer	2.8	4BUM	Schredelseker et al., 2014
mVDAC1	Mouse	Wildtype ATP complex	X-ray	Monomer	2.3	4C69	Choudhary et al., 2014
hVDAC1	Human	E73V	NMR	Monomer	–	5JDP	Jaremko et al., 2016
hVDAC1	Human	Wildtype	X-ray	Monomer	3.2	5XDN	Hosaka et al., 2017
hVDAC1	Human	Wildtype	X-ray	Monomer	3.1	5XDO	Hosaka et al., 2017

Arguably, a new level of insight into the structures of the VDAC superfamily emerged from recent papers on Tom40. Due to the close evolutionary relationship between VDAC and Tom40, data from both proteins taken together have enhanced the understanding of the VDAC structure. *In-organello*, cysteine and protease-accessibility mapping studies of Tom40 in the MOM clearly supported the 19-stranded VDAC model (Lackey et al., 2014) (see Figure 3C). More recently, cryo-electron microscopy (EM) of isolated and dimeric Tom40 complexes from *N. crassa* provided additional evidence for the accuracy of the 19-stranded VDAC structure, since it was used to construct the Tom40 model (Bausewein et al., 2017). This number of beta-strands also agrees with the pore diameter and the dimensions along the barrel. This EM study also provided insights into the natural dimerization mechanism and the flexibility of the internal N-terminal helix. Interestingly, the N-terminal helix in Tom40 can adopt two conformations, one of which is the bent conformation inside the barrel also observed in the VDAC structures, while the second conformation places the folded helix outside the channel. Dimerization of Tom40 is accomplished via the β1 and β19 strands in the same way as described by Bayrhuber et al. (2008) for VDAC1 and later confirmed for the VDAC2 structure from zebra fish (zVDAC2) (Schredelseker et al., 2014). Interface mutants based on the zVDAC2 structure further confirmed the crystallographic interface previously reported. Sequence comparison of ncTom40 and hVDAC1 shows that identical or highly conserved residues are located on the b1 and b19 strands (Bay et al., 2012 and our unpublished data).

## Ion permeation and gating of VDAC elucidated by molecular dynamics simulations

A wide range of simulation studies have addressed the question of VDAC dynamics and permeation. Molecular dynamics (MD) simulations based on the mVDAC1 (PDB-entry: 4C69) structure combined with a Markov state model showed the capacity to pass millions of ATP molecules per second via hundreds of different permeation pathways along a network of basic residues (Choudhary et al., 2014). Prior to this, MD and Brownian Dynamics studies on the mVDAC1 structure (PDB-entry: 3EMN) had established ion transfer pathways and the ion conductance and selectivity of VDAC, reporting a high level of agreement with experiment and illuminating the molecular basis of the pronounced anion-selectivity of VDAC (Lee et al., 2011; Rui et al., 2011). Poisson-Nernst-Planck and electrostatics calculations shed further light on the anion-selective permeation across VDAC and highlighted mutations that could reverse this selectivity to transform VDAC into a cation-selective porin (Choudhary et al., 2010). The crystal structure of murine mVDAC1 (PDB-entry: 3EMN) was also used to elucidate the energetic and kinetic basis of ATP and ADP translocation across the pore by umbrella sampling calculation as well as its molecular interaction with the cytoskeletal protein tubulin by ROSETTA-based protein-protein docking (Noskov et al., 2013). These computational findings characterized the binding between mVDAC1 and tubulin, which was previously reported from experimental work. This interaction is considered to be physiologically important for the gating of VDAC and, consequently, for the permeability of the MOM. Finally, two studies using a combination of solution or solid-state NMR and extended molecular dynamics simulations based on the mVDAC1 structure arrived at the conclusion that the beta-barrel structure of VDAC exhibits a particularly high flexibility (Villinger et al., 2010) and that, especially following a voltage-dependent dislodgement of the internal alpha-helix, a significant deformation of the barrel can explain subconductance states at greatly altered ion-selectivity, as previously described experimentally (Zachariae et al., 2012). In the latter study, ion conduction and selectivity was probed in fully open and semi-collapsed barrel states with the Computational Electrophysiology (CompEL) simulation technique (Kutzner et al., 2016). The role of the beta-barrel in VDAC and porin channel gating was confirmed in a range of experimental electrophysiology studies by Essen and colleagues (Grosse et al., 2014), which also characterized its dependence on the dynamics of the N-terminal helix (Mertins et al., 2014), and its flexibility in the membrane corroborated by further NMR studies (Ge et al., 2016). The subconductance states of VDAC were recently also addressed in a combined experimental and computational study using CompEL and single-channel electrophysiology (Briones et al., 2016), which confirmed the existence and further elucidated the conformation of cation-selective subconductance levels in VDAC. Furthermore, a recent review collating a wide array of experimental and computational data came to the conclusion that the beta-barrel collapse model is in agreement with the majority of experimental observations on VDAC including its lipid-sensitivity (Shuvo et al., 2016).

## Recent developments and future directions

For the last 10 years, research on VDAC has rested on high resolution structures, which clearly described the fold of VDAC-superfamily proteins. Recent investigations have therefore focused on the interpretation of physiological data by molecular dynamics, for example to explain functional changes such as the switch from anion- to cation-selectivity on the basis of structural dynamics as well as the decreasing channel diameter upon voltage application. Despite a wealth of such studies, and a remarkable level of new insight achieved, some basic physiological questions remain open. These include the structural basis for the initiation of the transition between high and low conductance states, clearly defined binding sites for nucleotides, and the molecular interactions formed with apoptosis-related proteins (see also Noskov et al., 2016). However, STED microscopy improved our understanding of VDAC-HK complexes formation under physiological conditions on the molecular level (Neumann et al., 2010). Most recent NMR and X-ray structures were not able to enhance our view on how VDAC is embedded into natural membranes, and the best images of natively enriched VDAC samples still come from early EM data and data from atomic force microscopy. Using Tom40 as a representative of the VDAC superfamily, two recent investigations clearly confirmed its specific fold under biological conditions. Although corroborative in terms of the VDAC-fold, the EM study of Tom40 in particular provided a genuine breakthrough for the field, as for the first time protein complexes in the MOM were structurally unveiled at 1 nm resolution (Bausewein et al., 2017).

## Further VDAC structures, which would help to understand its physiology as a channel connecting mitochondria with the cytoplasm:

- Structure and putative function of the closed or semi-open state
- Structure of metabolite (e.g., ATP/ADP, citrate) and drug complexes (e.g., erastin)
- Structure of VDAC3
- Structures of VDAC-ANT complexes
- Structures of VDAC in complex with pro-apoptotic proteins such as tBid, Bax, or Bak.

## Author contributions

KZ and UZ contributed to conception and design of the manuscript and wrote the manuscript.

### Conflict of interest statement

The authors declare that the research was conducted in the absence of any commercial or financial relationships that could be construed as a potential conflict of interest.
